# Perspective on Using Artificial Intelligence in Alcohol Research and Treatment: Opportunities and Ethical Considerations

**DOI:** 10.35946/arcr.v45.1.12

**Published:** 2025-11-26

**Authors:** David I.K. Moniz-Lewis, Megan Kirouac, Matison W. McCool, Frank J. Schwebel, Katie Witkiewitz

**Affiliations:** 1 Department of Psychology, University of New Mexico, Albuquerque, New Mexico; 2 Center on Alcohol, Substance use, And Addictions, University of New Mexico, Albuquerque, New Mexico

**Keywords:** alcohol, alcohol use disorder, addiction research, artificial intelligence, machine learning, large language models, research ethics, treatment ethics

## Abstract

**PURPOSE:**

There is an urgent need for novel and innovative advancements in alcohol research and treatment. Artificial intelligence (AI) is one such advancement that demonstrates promise, though not without important ethical concerns. This Perspective article examines current applications, ethical considerations, and future implications of integrating AI in alcohol research and treatment.

**SEARCH METHODS AND RESULTS:**

A nonsystematic narrative review of peer-reviewed, English-language studies of contemporary applications of AI (specifically, large language models and machine learning) in alcohol research and treatment was conducted in December 2024 through January 2025, using searches of the PubMed, Google Scholar, Web of Science, and PsycInfo databases. Search terms included the following: “alcohol,” “alcohol use,” “alcohol use disorder,” “drinking,” “treatment,” “screening,” “artificial intelligence,” “chatbot,” “large language models,” “research subject recruitment,” “research subject enrollment,” “electronic health records,” “support vector machine,” “neural network,” “black-box,” “random forest,” and “machine learning.” The article highlights studies that discussed the role of AI in bolstering recruitment and retention for human subjects research and in the application of advanced data analytic techniques for alcohol research. Further, the article examines studies that described current and prospective uses of AI in alcohol use disorder treatment, including assessment and identification of individuals who may benefit from treatment, and as an adjunct to evidence-based treatments for alcohol use disorder. Potential ethical concerns are highlighted alongside potential considerations for the safe and ethical use of AI across these domains.

**CONCLUSIONS:**

Based on the identified primary and secondary sources, this review highlights the promises of AI in advancing both alcohol research and treatment, and how its successful implementation is supported by careful and ongoing attention to potential risks. The reviewed studies provide evidence that robust data security protocols, comprehensive informed consent, and ongoing monitoring for potential biases and harms can allow for these tools to advance scientific understanding while improving access to effective and equitable care. However, the current literature and the potential solutions suggested in this Perspective have limitations due to the pace of AI advancements that continue to accelerate at an unprecedent rate.

**FUTURE DIRECTIONS:**

Future research can advance the field by explicitly testing and evaluating the utility of these tools in real-world contexts, their risks for bias and privacy violations, and their ethical integration into alcohol research and treatment.

**KEY TAKEAWAYS**
- Artificial intelligence (AI) holds significant potential for improving recruitment, retention, and data analysis in alcohol research using human participants.
- AI may also improve the effectiveness and accessibility of evidence-based treatments for alcohol use disorder (AUD).
- The empirical literature examining ways in which AI can be implemented in AUD treatment and alcohol research remains largely preliminary, highlighting the potential for future research that adequately addresses important ethical challenges.
- Ethical concerns, including data privacy, algorithmic bias, utility for diverse populations, and an overreliance on AI systems in clinical contexts, can be addressed to ensure equitable and effective implementation.
- Future advancements in AI, including the development of artificial general intelligence, could radically transform alcohol research and AUD treatment and thus may benefit from ongoing ethical oversight and adaptation of best practices.

Alcohol use disorder (AUD) is a significant public health challenge affecting millions of individuals globally.^1^ In the United States, the mortality associated with AUD and alcohol misuse continues to rise, with more than 140,000 deaths attributed to alcohol annually from 2015 to 2019, and an additional 25.5% increase in alcohol-related deaths from 2019 to 2020; further, more recent data indicate that more than 178,000 deaths are attributable to alcohol misuse per year.^2^–^4^ Despite the existence of evidence-based pharmacological, behavioral, and psychosocial treatments for AUD, significant social and structural barriers prevent many individuals from accessing treatment.^5^,^6^ Such barriers have contributed to a landscape where less than 10% of affected individuals who might benefit from AUD treatments ever receive care.^7^ These observations underscore the urgent need for innovative solutions that can improve access to evidence-based AUD treatment. Over the last decade, various novel technologies and digital innovations have emerged that have the potential to improve the identification of individuals who might benefit from AUD treatment while simultaneously increasing access to care. One such innovation that may offer unique promise is the use of artificial intelligence (AI).

AI is a broad term referring to computer systems designed to perform tasks that typically require human intelligence, such as algorithmic learning, adaptive reasoning, and complex decisionmaking.^8^ AI encompasses multiple subfields of technologies, such as supervised and unsupervised learning, reinforcement learning, and broader learning architectures. Machine learning is a common application of AI and often includes predictive machine learning (i.e., predictive models using structured data) and large language models (LLMs). Machine learning refers to computational algorithms that are designed to emulate human intelligence by learning from novel data, which can include mixture modeling approaches.^9^ Machine learning algorithms seek to continuously improve performance on various tasks (e.g., predictive modeling) without explicit human input. LLMs aim to process and generate human-like speech and text in response to human input.^10^ LLMs are currently capable of engaging in human-like conversations and writing, with recent evaluations demonstrating capacities comparable to those of graduate-level students.^11^ However, current AI technologies still differ from the theorized future development of artificial general intelligence (AGI)—an anticipated evolution of current AI that is expected to replicate or exceed human cognition across a broad range of cognitive tasks.^12^ This enhanced capacity could potentially enable AGI systems to learn, reason, and adapt to entirely new contexts without the constraints imposed by programming and training data. Although AGI to date remains hypothetical, generative AI (i.e., AI systems that can create original content, such as text, images, audio, or software code in response to user prompts) is already beginning to make notable impacts on alcohol research, treatment of AUD and alcohol misuse, and varied applications in other mental health and substance use disorder research and treatment.^13^–^18^ This article highlights the potential promises and challenges of using AI to advance alcohol research and treatment and provides an overview of how AI can improve access to equitable, effective, and compassionate care.

## Search Methods and Results

A nonsystematic narrative review of existing peer-reviewed studies on the use of AI in alcohol treatment and research was conducted in December 2024 through January 2025 using the PubMed, Google Scholar, Web of Science, and PsycInfo databases. Search terms included the following: “alcohol,” “alcohol use,” “alcohol use disorder,” “drinking,” “treatment,” “screening,” “artificial intelligence,” “chatbot,” “large language models,” “research subject recruitment,” “research subject enrollment,” “electronic health records,” “support vector machine,” “neural network,” “black-box,” “random forest,” and “machine learning.” Articles were deemed relevant by the authors if the article provided information on current and prospective uses of AI in alcohol research and treatment. The review focused on LLMs and machine learning and did not examine forms of AI beyond these, as these technologies appear to have been studied the most at this time. The references of the included articles were further reviewed to identify additional relevant articles. There were no constraints on year of publication. Inclusion criteria included peer-reviewed articles written in English and deemed relevant to the following topics: the use of AI in alcohol research, the use of AI in alcohol treatment, and ethical considerations for the use of AI in both domains. Two streams of sources were included: (1) primary empirical studies and (2) secondary sources (e.g., reviews, and commentaries). The latter were included because the current literature on AI in alcohol research and treatment is still in its infancy, with limited primary empirical studies. Indeed, many of the most important questions surrounding the use of AI (e.g., how to ethically employ this technology) are largely unanswered via primary empirical studies at present. Only gray literature (i.e., dissertations, theses, and other non-peer reviewed sources) was excluded from this review. In total, 28 sources were reviewed. All authors participated in the search and review process.

## Using AI in Alcohol Research

### Recruitment and Retention of Study Participants

Use of AI in recruitment and retention of human study participants has greatly increased since 2020,^19^ with various aspects of recruitment (e.g., participant identification, advertisement, screening, and data collection) expanded by AI advancements. Data mining, natural language processing, and practical tools, such as automated alerts to study personnel have all been used to streamline recruitment processes.^20^ Although these technologies can be associated with additional costs to researchers, they also may improve efficiency of research procedures (e.g., saving personnel time needed for reviewing medical records, posting flyers, conducting in-person or phone-based screens).^19^ Moreover, targeted recruitment approaches that utilize ethical data mining and adaptive machine learning strategies may allow researchers to reach populations who are historically underrecruited and/or geographically isolated; this is particularly important given that only approximately 2% of the population participates in clinical research.^21^

Once participants have been recruited, AI may also be used to help with participant retention, although the use of AI in this area has been much less studied than its use for recruitment.^19^ Abiodun and colleagues have proposed a framework by which researchers can leverage AI technologies, such as artificial neural networks and support vector machines, to assist in decision-making on whether participants can be allowed to proceed in a clinical trial.^22^ It is probable that with careful human oversight,^23^ AI could also be used to enhance retention in clinical trials via automated tools to support patient engagement, such as sending newsletters and birthday/holiday/thank you cards with study-specific branding and information to acknowledge ongoing participation.^24^ Digital tools that rely on AI may also aid in enhancing engagement in study procedures in general,^25^ and machine learning could be used to predict participants’ premature dropout from research.^26^ For example, Lopez and colleagues used a logistic regression classifier to predict premature discontinuation of medications for opioid use disorder, and similar approaches may help to predict premature dropout from alcohol research.^26^ However, there is a need for more research on each of these AI applications to improve retention and optimize the equitable inclusion of participants from varied backgrounds.^25^

### Data Science and Analysis

Once participants have been recruited and retained in alcohol research, the analysis of participant data can be greatly enhanced through advanced machine learning methodologies. Over the past decade, alcohol researchers have used machine learning models to examine a range of complex questions— including risk and protective factors for the development of problems related to alcohol use,^27^,^28^ remission from AUD,^28^ risk of adverse events (i.e., suicide-related events, death),^29^ risk of returning to drinking following a period of abstinence^30^—to predict alcohol treatment seeking and treatment goals^31^,^32^ and to predict drinking behavior and outcomes in alcohol-related clinical trials.^33^–^37^ For example, Kinreich and colleagues used a form of machine learning called least absolute shrinkage and selection operator (LASSO) to use patient demographic data to predict the likelihood of developing AUD.^27^ Similarly, Afzali and colleagues compared seven different forms of machine learning (e.g., LASSO, support vector machines) to identify predictors of alcohol use.^26^ Although these advanced machine learning algorithms offer many applications to alcohol research, they vary widely in their algorithms, purposes, and suitability for different research questions and can thus be categorized based on these criteria. This can be illustrated by comparing models that provide insights into how feature variables interact to predict outcomes versus models that offer little insight into predictors (e.g., “black-box” models—machine learning algorithms in which the connections between the predictors and outputs are either too complex for human interpretation or completely unknown).^38^

Some machine learning models use tree-based algorithms— a method that uses branching to iteratively split the data into progressively smaller groups based on a predictor. These approaches can be used to identify predictors that best split the data into groups for classification problems (classification trees), such as predicting who has AUD and who does not, or to minimize the variance in the outcome for regression problems (regression trees), such as predicting outcomes of AUD treatment.^39^ Tree-based algorithms include recursive partitioning (sometimes referred to as decision trees), in which data are repeatedly divided into subsets according to the predictor that provides the best separation, and conditional inference trees, which use statistical hypothesis testing to determine each branch of the tree.^40^

However, these algorithms are vulnerable to overfitting (i.e., modeling noise in training data such that performance looks strong in the training sample but degrades when provided new data sets), which limits model generalizability. Ensemble machine learning algorithms, such as random forests, attempt to decrease overfitting by building numerous trees (potentially thousands) with random subsets of predictors and providing a metric for variable importance (i.e., how much a given variable improves model fit). Other ensemble methods incorporate black-box methods, such as gradient boosting, which iteratively builds new trees while correcting errors in the previous tree. Although each of these approaches can provide insight into the key predictors of an outcome, the selection of important predictors and splits in a tree can be driven by high multicollinearity between predictor variables. This means that predictors are highly correlated, making a determination of their independent effects on the outcome more challenging and potentially leading to unstable estimates, which may consequently complicate interpretability. However, multicollinearity is not only relevant to machine learning, but also affects traditional statistical models, often leading to unstable estimates which lead to model nonconvergence.^39^,^41^

Whereas single tree-based algorithms tend to focus on interpretability of the results, black-box methods—such as neural networks, support vector machines, and gradient boosting—focus on examining complex interactions among the predictor variables that most improve outcome prediction. Black-box methods can handle a large number of predictors and, due to their focus on finding complex nonlinear interactions, may be superior to tree models regarding outcome prediction.^42^ However, these models provide little to no interpretability,^43^ and attempts to provide insight into which combinations of predictors or interactions are most important still suffer from similar issues as tree-based models regarding multicollinearity and assigning unique importance to correlated predictors.^38^

Traditional variable-importance methods (e.g., linear regression estimates) share these limitations but remain the most commonly used option for interpretability tools. Recent developments in explainable AI, or understanding the black box through new methods of measuring variable importance, attempt to explain predictor relationships at the individual level and aggregate those importance metrics across people, as opposed to across predictors. Although these approaches also still suffer from having to disentangle correlated predictors,^44^ these developments suggest that black-box models are increasingly useful for analyses focused on both strong prediction and meaningful interpretability. Moreover, new tools are expanding access to these models to researchers outside of computer science.^45^

In sum, machine learning models may be better suited for analyzing high-dimensional data and hypothesis generation than for hypothesis testing. Specifically, these machine learning methods can flexibly analyze complex, nonlinear relationships; handle high feature count; detect interactions between variables; and model complex interactions.^46^ However, the models are only as good as the data they are trained on. When deployed in the real-world, models that are trained on large data sets can have unintended consequences.^43^ For example, in health care research, machine learning models tend to be trained on imbalanced data sets regarding race and other social determinants of health. This may result in models that predict favorable outcomes and paths for White individuals and those from higher socioeconomic status, but yield poorer outcomes and lower accuracy for individuals with minoritized identities.^47^

## Using AI in Alcohol Treatment

The previous section largely focused on the use of AI in big-data contexts; however, there is also a considerable expansion of AI in clinical delivery research. To date, the implementation of AI in alcohol treatment has demonstrated unique promises with important ethical considerations across two key domains: (1) assessment and identification of individuals with alcohol misuse and AUD, and (2) provision of evidence-based psychotherapy. Understanding the current capabilities and constraints of AI across these domains is important for the ethical and effective integration of these tools into clinical practice.

### AI in Alcohol Use Assessment and Screening

Identifying individuals who meet criteria for AUD or misuse alcohol is an important first step in improving access to evidence-based care and ameliorating the harm resulting from alcohol misuse. Recent research has demonstrated promise in the capacities of AI technologies, particularly machine learning algorithms, to efficiently identify individuals at risk for AUD via mass screening of electronic health records.^48^,^49^ For example, Afshar and colleagues used natural language processing and supervised machine learning to identify people at risk of alcohol misuse based on data in patient health records.^47^ Similarly, Vydiswaran and colleagues used a rule-based natural language processor to identify risk factors for alcohol use from clinical notes in patients’ electronic health records.^48^ Beyond chatbots, mobile health platforms can also use reinforcement learning algorithms to deliver personalized just-in-time adaptive interventions that trigger support when potential risks are detected in daily life (e.g., high levels of craving).^50^,^51^ Further, tools such as Lyssn AI, developed by Atkins and colleagues, build upon decades of research supported by the National Institute on Alcohol Abuse and Alcoholism on motivational interviewing to automatically code motivational interviewing sessions and provide immediate fidelity feedback (i.e., how well the provider adhered to motivational interviewing principles and techniques) to support providers’ delivery of the intervention.^52^ Of particular promise are algorithms that combine multiple biomarkers and demographic variables to identify individuals who likely meet criteria for AUD and may not yet be receiving care.^53^–^55^ For example, Kamarajan and colleagues used random forest plots to classify risk for AUD via a combination of neuropsychological test scores and functional magnetic resonance imaging data.^52^ Although still largely preliminary, such findings allude to a future where large medical systems may rapidly identify individuals who may benefit from evidence-based alcohol treatment.

However, it is necessary to acknowledge and account for the potential inherent biases within these tools. The current effectiveness of any AI tool is predicated on the quality of its training data. Without careful curation and thorough analysis, the data used to train these tools may inadvertently reinforce existing biases and health disparities, and thus ultimately undermine their ability to serve all patient populations and improve health equity.^56^ For example, recent work has shown that among U.S. veterans, Black and Hispanic veterans are more likely than White veterans to receive an AUD diagnosis despite reporting similar levels of alcohol consumption, with this effect holding even after controlling for potential confounders.^57^ AI diagnostic tools trained on such data risk perpetuating these same disparities by potentially overdiagnosing racial and ethnic minority patients with AUD. Thus, concerted efforts are needed to advance these technologies in a manner that incorporates diverse, high-quality training data and includes rigorous and continual evaluation in real-world clinical settings.^56^,^58^

### AI in Alcohol Use Treatments

In parallel to advancements in AI-driven screening and identification, advancements have emerged in the use of LLMs to enhance and improve alcohol treatment. The proliferation and popularity of commercially available LLMs have fueled a concurrent growth in the application of LLMs for mental health disorders, primarily through conversational agents (i.e., “chatbots”). Although many of these chatbots are novel and require further evaluation, current research suggests that these tools may be readily able to increase access to evidence-based interventions beyond traditional clinical settings. Recent research has demonstrated that AI chatbots could effectively deliver core ingredients of gold standard, evidence-based AUD treatments, such as cognitive behavioral therapy and motivational interviewing.^59^–^63^ For example, Boustani and colleagues developed an LLM to deliver motivational interviewing,^57^ whereas Prochaska and colleagues developed an LLM to support individuals in reducing their alcohol use via cognitive-behavioral principles.^59^,^60^ Further, research has demonstrated that these tools could not only deliver the core components of these treatments but may also foster critical “common factors,” such as empathy and therapeutic alliance.^63^–^65^ However, recent work also has suggested that the optimal, and perhaps most ethical, use of AI tools in AUD treatment is to complement, not substitute, direct human clinical intervention.^66^ This is in part due to data privacy and security concerns inherent to the use of AI in clinical settings.^67^ Additionally, there is increasing concern about the effectiveness of LLMs to identify and intervene in high-risk situations, such as in cases of imminent risk of harm to self or others.^68^ The latter concern presents a twofold ethical dilemma whereby AI tools may lack the capacity to identify high-risk situations, or conversely, they may become over-relied upon and thus undermine clinician judgment and patient testimony.^69^ Nonetheless, with careful and continuous evaluation, AI offers promise in enhancing and expanding access to evidence-based psychotherapy for AUD, which is crucial, considering that less than 10% of people who need AUD treatment currently receive it.^7^,^70^

## Maximizing Benefits and Reducing Ethical Concerns

The ethical integration of AI into AUD treatment and research may benefit from a commitment to ongoing evaluation to ensure that patient autonomy and well-being remain central to its application. Numerous ethical dilemmas may arise from the use of AI in clinical settings, most prominently concerns around the perpetuation of bias and health disparities, data safety and privacy, and efficacy among high-risk populations.^56^,^67^,^69^ Nonetheless, this review of the current literature has highlighted key considerations and potential solutions that alcohol researchers and clinicians can employ to maximize the benefits of these novel technologies while reducing potential harms.

Central to the use of AI in alcohol research are training data quality, data privacy and security, and the need for robust replication before allowing AI-informed research to influence policy and clinical practice. Ensuring data quality may include attention to the sociodemographic composition of training data for machine learning models, especially for models directly related to patient care. Future studies designed to evaluate machine learning tools would benefit from recruiting individuals that are balanced across a range of sociodemographic variables. Balanced data sets allow for more nuanced predictions and reduce biases that may disproportionately affect underrepresented groups. Further, most machine learning applications in AUD research to date have been exploratory; as such, findings would benefit from rigorous replication to verify their validity before influencing practice. Additionally, alcohol researchers at academic and research institutions often rely on cutting-edge developments of AI in the private industry, and many researchers are likely to outsource their use of LLMs to private companies catering their AI for research purposes. However, private companies are not beholden to the same ethical guidelines as most researchers, and some companies have a history of using copyrighted material, ambiguous user agreements, and questionable data management practices.^71^,^72^ Consequently, alcohol researchers using AI tools developed in the private industry must rigorously evaluate these tools to ensure they adhere to ethical standards. Other researchers are making efforts to develop secure AI tools to use for treatment.^73^ In each of these scenarios, researchers can allow open access to any AI tools that are being developed with private or public funding, such that the field can build on prior work and focus on replication of findings. This ensures generalizability of scientific findings across various settings and populations. Finally, data sharing with globally unique identifiers or similar, secure participant identifiers may also be beneficial to prevent potential confounds related to having the same individuals participate in multiple studies that use AI for recruitment.

The ethical integration of AI tools into alcohol treatment may best be supported by thoroughly evaluating their efficacy, risks of bias, and fidelity to empirically support practices before employing them in clinical settings.^74^,^75^ This can include validation that these tools perform reliably across sociodemographically diverse populations and clinical scenarios (e.g., for individuals with different severity of AUD).^56^ Further, a clear and accessible informed consent process is important so that patients are aware of the capabilities, limitations, and risks of using AI tools to address alcohol use, whether as a standalone intervention or as an adjunct to ongoing clinical care.^76^ Such an informed consent process can ensure that patients are empowered to make informed decisions about their use of the AI instrument and the use of the data they provide to it. Additionally, any health care system or provider offering such tools can establish systems for continuously monitoring and evaluating its impact on patient outcomes to identify potential biases in algorithms and ensure that the tool does not itself induce unintended harmful effects (i.e., iatrogenic effects). Such measures will require considerable infrastructural support, such as insurance reimbursement for asynchronous contact by treatment providers and additional tools that allow providers or health care systems to receive data directly.^67^ Clear a priori protocols for human intervention are also important, particularly when individuals using AI chatbots or similar tools present imminent risks to themselves or others. Furthermore, there is a need to establish and regularly evaluate robust data security frameworks to protect patient privacy and safeguard sensitive data.^67^ Lastly, implementation strategies that prioritize safety, accessibility, and equity can ensure that these tools aid in closing the AUD treatment gap rather than exacerbating it.

## Conclusions

This review has highlighted the promises of AI, specifically machine learning and LLMs, to improve alcohol research and treatment by advancing recruitment and retention for research, predicting alcohol misuse, screening electronic health records, and serving as an adjunct to evidence-based psychotherapy. However, these technologies, and the research reviewed here, are relatively early in development. Thus, there is a need for further evaluation of these tools before drawing more concrete conclusions about their utility in alcohol research and treatment. Nonetheless, the existing literature reviewed here highlights strengths of these tools in scalability, speed, and accessibility of both treatment and research. This literature further highlights important limitations, including biases within these technologies, privacy and security concerns, and a lack of research on best implementation practices of these tools in community and treatment agencies.

Another limitation is that the literature reviewed and the potential solutions suggested in this Perspective are based on the current AI landscape and focused on a small subset of AI technologies. The review did not comprehensively assess all forms of AI; rather, its scope was limited to the use cases of machine learning and LLMs in the existing literature on alcohol treatment and research. Further, the review focused on the use of AI in alcohol research and treatment and did not consider notable advancements occurring across other fields, including substance use disorders more broadly and general medicine.^77^,^78^ AI technologies are rapidly advancing at an unprecedent rate; accordingly, the potential solutions and findings reviewed here may soon prove obsolete.

## Future Directions

Over the last decade, the capacities of AI have advanced exponentially, and prospective novel tools such as AGI have the potential to radically alter the current landscape. Thus, the potential solutions offered in this Perspective can only serve as a current view on the ethical use of AI in alcohol research and treatment based on an overview of extant literature. Future research can advance the field by explicitly testing and evaluating the utility of these tools in real-world contexts, their risks for bias and privacy violations, and in what contexts these tools are most appropriate and least likely to unintentionally cause harm. No matter what the future advancements of these technologies have in store, the ethical integration of current and future AI tools into alcohol research and treatment will be an important consideration. As these technologies advance, it is important for potential solutions to their implementation to parallel these advancements to ensure that the field of alcohol research and treatment remains focused on prioritizing patient autonomy, well-being, and health equity.
